# Occurrence of Antibodies Anti‐*Toxoplasma gondii* in Captive Non‐Human Primates From Pernambuco State, Brazil

**DOI:** 10.1111/jmp.70099

**Published:** 2026-08-06

**Authors:** Marcio André Silva, Hilda Fátima Jesus Pena, Herbert Sousa Soares, Dênisson Silva Souza, Solange Maria Gennari, Jean Carlos Ramos Silva

**Affiliations:** ^1^ Post Graduate Program in Veterinary Medicine, Department of Veterinary Medicine Universidade Federal Rural de Pernambuco Recife Brazil; ^2^ Parque das Aves Foz do Iguaçu Brazil; ^3^ Brazilian Institute for Conservation Medicine (Tríade) Recife Brazil; ^4^ Department of Preventive Veterinary Medicine and Animal Health, School of Veterinary Medicine and Animal Science Universidade de São Paulo São Paulo Brazil; ^5^ School of Veterinary Medicine Universidade Santo Amaro São Paulo Brazil; ^6^ Indepedent Veterinarian Recife Brazil; ^7^ School of Veterinary Medicine Centro Universitário Max Planck—UniMAX Indaiatuba Brazil; ^8^ School of Veterinary Medicine Centro Universitário de Jaguariúna—UniFAJ Jaguariúna Brazil

**Keywords:** captivity, MAT, Platyrrhini, serology, toxoplasmosis, wildlife

## Abstract

Wildlife captive populations are important tools for threatened species conservation, and early identification of diseases must be prioritized. In this study, we test for the presence of anti‐*Toxoplasma gondii* antibodies in non‐human primates at a Zoo from Pernambuco State, Brazil. A Modified Agglutination Test (MAT ≥ 25) was carried out on serum samples from 28 individuals of nine species ( *Ateles chamek* , *Ateles marginatus* , *Ateles paniscus* , *Chlorocebus aethiops* , *Papio anubis* , *Papio hamadryas* , *Lagothrix lagotricha* , *Sapajus flavius* , and *Sapajus libidinosus* ) from two parvorders (Catarrhini and Platyrrhini). Among the tested animals, 19 (67.85%) from eight different species were seropositive, and only one *S. flavius* developed clinical toxoplasmosis. A statistical association was observed between age and presence of *T. gondii* antibodies, with adults and geriatric animals showing higher (*p* < 0.05) association than young ones. Local of born (in the zoo or out of the zoo), parvorder, and gender were not significantly associated (*p* > 0.05).

## 1 Introduction

Primates are a mammalian order found on all continents except Australia. They are divided into three large groups: prosimians (Strepsirrhini), Old World primates (Catarrhini), and New World primates (Platyrrhini); the last group occurring exclusively in Central and South America [1]. In Brazil, there are 139 known primate taxa (species and subspecies), many of which are endemic. Among these, 34 are considered threatened with extinction, and 14 lack data to categorize the degree of threat they are facing, according to the Brazilian Primatology Center of the Chico Mendes Institute for Biodiversity Conservation [1].

Maintaining safe populations of wild animals under human care is an important tool for species conservation, especially for the most threatened taxa [2]. Therefore, early identification of pathogens that can lead to reproductive failure or cause death is important to successfully breed wild animals in zoos and other institutions that work with ex situ wildlife management [3].

Among these pathogens, the protozoan *Toxoplasma gondii* causes reproductive failures including abortions and is almost always fatal to New World primates and Australian marsupials [3, 4, 5]. However, serological studies of the infection in live Neotropical primates are still few [6, 7, 8, 9, 10], and most toxoplasmosis‐related cases are through *post‐mortem* diagnosis, necropsy findings, and complementary tests, such as immunohistochemistry, histopathology, and detection of the agent by molecular biology in necropsy specimens [11, 12, 13, 14, 15].

Given the possible seriousness of infection by this agent in Neotropical primates and the scarce literature on the seroprevalence of anti‐*T. gondii* antibodies in these animals, the aim of this study was to carry out a serological survey of *T. gondii* infection in non‐human primates kept at the Parque Estadual Dois Irmãos Zoo, which carries out important conservation work for these species in northeastern Brazil.

## 2 Materials and Methods

This study was carried out at the Parque Estadual Dois Irmãos Zoo (PEDI Zoo), Recife Municipality, Pernambuco State, northeastern Brazil (8°7′30″ S, 34°52′30″ W). All procedures were carried out through the internal preventive medicine program of the Institution and strictly respected humane care in the handling of the animals involved, with an experimental protocol approved by the Ethics Committee for Animal Use in Research from Universidade Federal Rural de Pernambuco (CEUA‐UFRPE, protocol n° 109/2014), as well as authorization from the Parque Estadual Dois Irmãos (n° 216/2014) and the Biodiversity Authorization and Information System from Chico Mendes Institute for Biodiversity Conservation (SISBIO—ICMBio, authorization n° 37855‐1). The authors confirm that the ethical policies of the journal, as noted on the journal's author guidelines page, have been adhered to and that Parque Estadual Dois Irmãos zoo was recently accredited in best practices of animal welfare by Associação de Zoológicos e Aquários do Brasil (AZAB).

Serum samples from 28 non‐human Neotropical primates were obtained from individuals from nine species, six from the Parvorder Platyrrhini ( *Ateles chamek* , *Ateles marginatus, Ateles paniscus, Lagothrix lagotricha, Sapajus flavius*, and *Sapajus libidinosus* ) and three of the Parvorder Catarrhini (*Chlorocebus aethiops, Papio anubis*, and *Papio hamadryas* ).

Among these 28 individuals, six were tested multiple times; one *S. libidinosus* and four *S. flavius* had blood samples collected and analyzed twice, and one *S. flavius* had serum examined three times. The interval between those tests was a minimum of three months and a maximum of six months, according to the convenience and needs of the Institution.

Samples were taken by venipuncture of the saphenous, femoral, or caudal veins. The procedure was carried out with all animals under pharmacological restraint by injecting them with 10 mg/kg of ketamine hydrochloride combined with 0.5 mg/kg of midazolam, administered intramuscularly, after physical restraint with a hoist [16, 17]. Sampling was done by convenience, according to the need for pharmacological restraint of the animals established by the technical team of the zoo between 2014 and 2016. During this period, the animals were monitored for clinical signs suggestive of toxoplasmosis, as described by Dubey [4]. Blood samples were obtained without anticoagulant and centrifuged at 1500 *g* for 5 min to obtain serum, which was stored at −20°C in polypropylene microtubes [4, 8].

The Modified Agglutination Test (MAT) was used to test for IgG anti‐*T. gondii* antibodies, using formalin‐inactivated tachyzoites as the antigen, as described by Dubey and Desmonts [18]. A dilution of 1:25 [4] was used as cut‐off point and concomitant tests were carried out with previously known positive and negative mouse controls. Positive samples were serially diluted until the final reaction titer was reached [4]. The antigen used to perform the MAT was kindly provided by Dr. Jitender P. Dubey (United States Department of Agriculture, Beltsville, Maryland, USA).

The chi‐square test and G‐test of independence [19] were used to assess associations between the variables sex, age, birthplace at PEDI Zoo, and parvorder—Platyrrhini and Catarrhini, corresponding to New World and Old‐World primates, respectively—and the serological results, classified as seropositive or seronegative. A significance level of 5% was adopted (*p* ≤ 0.05). The analyses were performed in the R environment [20], using the RStudio interface and the *DescTools* package [21]. The data were analysed using all animals (New World and Old‐World primates, *n* = 28) and with only New World primates included (*n* = 24).

## 3 Results

Among the nine species studied, all except *L. lagotricha* , which was represented by a single individual, had at least one specimen positive for anti‐*T. gondii* antibodies (Table 1). Among the 28 evaluated animals, 19 (67.85%) were seropositive for *T. gondii*, with antibody titers ranging from 25 to 51 200. Of the six animals sampled more than once, four (one *S. libidinosus* and three *S. flavius* ) were seropositive from the first sample collection onward and maintained their serological status in subsequent tests, despite variations in antibody titers (Table 2).

**TABLE 1:** Occurrence of antibodies anti‐*Toxoplasma gondii*, by modified agglutination test (MAT > 25), in different primate species. Recife, Pernambuco State, Brazil.

Species	Common name	N. of animals		%
		Positive	Tested	
*Ateles chamek*	Black‐faced Spider Monkey	2	3	66.7
*Ateles marginatus*	White‐cheeked Spider Monkey	1	1	100
*Ateles paniscus*	Guiana Spider Monkey	2	2	100
*Chlorocebus aethiops*	Grivet Monkey	1	1	100
*Lagothrix lagotricha*	Woolly Monkey	0	1	0
*Papio anubis*	Olive Baboon	1	1	100
*Papaio hamadryas*	Hamadryas Baboon	2	2	100
*Sapajus flavius*	Blonde Capuchin	5	8	62.5
*Sapajus libidinosus*	Bearded Capuchin	5	9	55.5
Total		19	28	67.85

**TABLE 2:** *Toxoplasma gondii* antibody titers in serum samples, by Modified Agglutination Test (MAT ≥ 25), and identification of positive animals by species, sex and date of sample collection—Recife, Pernambuco State, Brazil.

Species	Common name	Sex	Identification	Antibody titers			
				02/2014	05/2014	11/2014	03/2015
*Ateles chamek*	Black‐faced Spider Monkey	M	#21147	3200	—	—	—
*Ateles chamek*	Black‐faced Spider Monkey	F	#21528	12 800	—	—	—
*Ateles marginatus*	White‐cheeked Spider Monkey	F	#21456	1600	—	—	—
*Ateles paniscus*	Guiana Spider Monkey	F	#19600	25 600	—	—	—
*Ateles paniscus*	Guiana Spider Monkey	F	#19374	25 600	—	—	—
*Chlorocebus aethiops*	Grivet Monkey	F	#27494	6400	—	—	—
*Papio anubis*	Olive Baboon	F	#40936	800	—	—	—
*Papaio hamadryas*	Hamadryas Baboon	M	#21582	25	—	—	—
*Papaio hamadryas*	Hamadryas Baboon	F	#25153	12 800	—	—	—
*Sapajus flavius*	Blonde Capuchin	F	#83264	6400	—	—	—
*Sapajus flavius*	Blonde Capuchin	M	#10487	51 200	—	—	—
*Sapajus flavius*	Blonde Capuchin	M	#03753	25 600	12 800	12 800	—
*Sapajus flavius* ^a^	Blonde Capuchin	M	#58516	25 600	12 800	—	—
*Sapajus flavius*	Blonde Capuchin	F	#50494	12 800	25 600	—	—
*Sapajus libidinosus*	Bearded Capuchin	F	#05215	12 800	—	—	—
*Sapajus libidinosus*	Bearded Capuchin	F	#05203	25 600	—	—	—
*Sapajus libidinosus*	Bearded Capuchin	M	#05216	12 800	—	—	—
*Sapajus libidinosus*	Bearded Capuchin	M	#05211	12 800	—	—	—
*Sapajus libidinosus*	Bearded Capuchin	M	#05214	—	—	6400	6400

*Note:* — blood sample was not collected.

Abbreviations: F, female; M, male.

^a^ Showed clinical signs compatible with toxoplasmosis.

The statistical analyses of the variables sex, age, birth at PEDI Zoo and Parvorder (Table 3) showed a significant association only with age, with a higher number of seropositive animals in the adult and geriatric groups than in the young group (*p* = 0.014). The same statistical analysis was performed using only New World primates and showed similar results (*p* = 0.026).

**TABLE 3:** Statistical analyses results regarding the variables sex, age, birth at Zoo PEDI and Parvorder.

Variable	Category	Total no. of animals	No. of seropositive animals (%)	*p*
Situation 1 (with all primates—*n* = 28 animals)				
Sex	Male	12	8 (66.7)	
	Female	16	11 (68.8)	1.000
Age	Young	2	0 (0)	
	Adult	19	12 (63.2)	
	Geriatric	7	7 (100)	**0.014**
Born at PEDI Zoo	Yes	2	0 (0)	
	No	26	19 (73.1)	0.095
Parvorder	Platyrrhini	24	15 (62.5)	
	Catarrhini	4	4 (100)	0.273
Situation 2 (without Parvorder Catarrhini – *n* = 24 animals)				
Sex	Male	11	7 (63.63)	
	Female	13	8 (61.53)	1.000
Age	Young	2	0 (0)	
	Adult	17	10 (58.82)	
	Geriatric	5	5 (100)	**0.026**
Born at PEDI Zoo	Yes	2	0 (0)	
	No	22	15 (68.18)	0.095
Parvorder	Platyrrhini	24	15 (62.5)	0.130

Only one male *S. flavius* ID#58516 (Table 2), which had been seropositive since the first collection, presented clinical signs compatible with toxoplasmosis three months after its last blood sample collection. The animal showed apathy, prostration, acute pneumonia and evolved to death in less than 24 h. The clinical signs began 48 h after the animal was kicked out from the troop due to disputes over dominance. The clinical signs and the main necropsy findings (enteritis, peritonitis, pulmonary edema, hepatosis and splenomegaly) were suggestive of acute toxoplasmosis [4, 11, 12, 13, 14, 17, 22, 23]. Immunohistochemistry, to confirm the necropsy findings, was not performed due to insufficient tissue availability. Tissue samples from the brain, heart, skeletal muscle and diaphragm were collected from this individual and submitted for molecular diagnosis by Polimerase Chain Reaction (PCR), according to Silva et al. [24]. A positive result was detected only in the diaphragm sample.

Also, during the monitoring period, the two *A. paniscus* and the two *P. hamadryas* included in the present study died, but none of them showed clinical signs or macroscopic alterations compatible with toxoplasmosis. The other animals remained free of any clinical signs that could be associated with *T. gondii* infection throughout the whole study period.

## 4 Discussion

According to Ferreira et al. [8], who analyzed serum from primates of the genus *Sapajus*, from five institutions located in northeastern Brazil, the main risk factors associated with *T. gondii* infection were advanced age and consumption of raw meat. Except for the predominantly frugivorous (and occasionally insectivorous) *Ateles*, the species evaluated in this study are all omnivores that consume small vertebrates [23, 25]. At PEDI Zoo, the diet of primates does not include raw meat, and their protein source comes from commercial extruded primate feed and boiled eggs. However, according to staff reports, insects and small vertebrates are present in the enclosures and the primates feed on them. In addition, synanthropic cats and wild felids, such as ocelots ( *Leopardus pardalis* ), also use the forest area in which the zoo is located, that could be a source of *T. gondii* infection [10]. The only variable statistically associated with seropositivity was age, with a higher number of seropositive adult and geriatric animals, supporting the previous findings [8].

As for the origin of the animals used in this study, only two were born at PEDI Zoo, two were of unknown origin and 25 came from other institutions (19 animals from Wild Animal Screening and Recovery Centers, five from zoos and one from a Conservation Breeding Facility). The pair of bearded capuchin monkeys ( *S. libidinosus* ), born at the zoo, remained negative throughout the whole study period. In the other primates, the time of infection cannot be assessed, as there is no way of knowing whether the infection was acquired before they entered the PEDI Zoo in their places of origin.

Asymptomatic infections seem to be the most frequent clinical presentations in humans and Old‐World primates infected with *T. gondii* [4, 5]. However, in New World primates, hyperacute and fatal clinical manifestations are most frequently described [3, 4, 5, 11, 12, 13, 14, 15, 23, 26, 27, 28].

Ferreira et al. [8] described 82.8% (111/134) of primates of *Sapajus* genus, from four different species, positive for anti‐*T. gondii* antibodies without showing clinical signs; and the primates also came from Wild Animal Screening Centers (*n* = 5) and zoos (*n* = 2) located in northeastern Brazil. In the same region, at Aracaju Zoo, Sergipe State, Pimentel et al. [24] found two *Sapajus* species, with 3 of 14 (21.4%) *S. libidinosus* and 3 of 4 (75%) *S. xanthosternus* reagents for *T. gondii* antibodies. In both studies [8, 29], antibody titers > 500 were found, as observed in practically all animals of the present study, with only one *P. hamadryas* showing a titer of 25 and all the others presenting a titer > 800.

Discrepancies in anti‐*T. gondii* antibody titer levels and high titer values in some individuals cannot be considered exclusively as indicators of recent infection, active infection, or the virulence capacity of *T. gondii* strains, as these variations and titer values are also linked to characteristics of the host species or to individual variation in the response capacity of the immune system of each organism [4, 5, 8, 30, 31]. In this study, the antibody titer variations found among different animals, or even among tests of the same individuals at different times, are probably related to individual immunological responses.

Studies observing other Neotropical primate species reacting to anti‐*T. gondii* antibodies are even scarcer, possibly due to fact that infection in these animals is almost always acute and fatal, with some genera being more susceptible, such as *Samiri*, *Alouatta* and all genera of the Callitrichidae family [3, 4, 17, 30]. In a study of free‐living wild animals in French Guiana, Thoisy et al. [31] found that 4/50 *Alouatta seniculus* were seropositive for *T. gondii* infection, without showing any clinical signs. In a similar study carried out in Paraná State, Brazil, Garcia et al. [6] identified asymptomatic seropositivity in 3/17 *Alouatta caraya* and in 13/43 *Sapajus apella* . Vitaliano et al. [30] also identified seropositivity in one *S. apella* from São Paulo State, Brazil, but this study does not mention whether there were clinical signs of toxoplasmosis in this animal.

Most studies involving the diagnosis of *T. gondii* infection in *Saimiri* species and in members of the Atelidae and Callitrichidae families report necropsy findings resulting from deaths by acute and fatal toxoplasmosis [5, 11, 12, 13, 14, 15]. Few studies reported asymptomatic seropositivity in primates of Atelidae family: two of three *Ateles paniscus* , kept in captivity in a zoo in Pará State, northern Brazil, with an antibody titer of 3200 [9]; two *Ateles geoffroyi* , kept in zoos in Mexico City, Mexico, both with a titer of 3200 [32], and two *Ateles marginatus* and two *Ateles chamek* in a zoo in Bauru, São Paulo State, Brazil, with titers varying from 25 to 200 [10].

Two studies describing *T. gondii* infection in woolly monkeys of the genus *Lagothrix* reported deaths from hyperacute infections, with a post‐mortem diagnosis [11, 33]. In these studies, serological tests were carried out on samples from the individuals that died and healthy animals, and anti‐*T. gondii* antibodies were only identified in the sera of the former [32]. In the present study, the only woolly monkey tested was negative.

Catão‐Dias et al. [23], in an extensive literature review of studies on *T. gondii* infection in several New World primate species, reported that infections in animals of genus *Lagothrix* tend to be fatal, with no reports of living seropositive animals in the compiled studies. However, for primates of the genera *Sapajus/Cebus*, the prevalence of seropositive animals among apparently healthy captive individuals ranged from 28.7% to 79%, depending on the species analyzed. In the same review, primates of genus *Ateles* showed prevalence rates ranging from 0% to 100%, depending on the species or study; however, because of the small number of individuals tested, the authors chose to exclude them from comparisons with species showing higher rates of asymptomatic carriage.

In the present study, the results obtained for the different primate genera were similar to those described by Catão‐Dias et al. [23] in their review, including the low number of individuals of genus *Ateles*, which precluded the identification of a standard serological response pattern for primates of this genus. In another study conducted in Brazil, Silva et al. [10] reported percentages of seropositive animals similar to those found in the present study; they also reported a low number of individuals of the genus *Ateles*, suggesting that Brazilian zoos maintain small populations of these primates.

The occurrence of clinical toxoplasmosis depends on many factors, ranging from parasite strain and virulence to host species susceptibility and the occurrence of stressful or immunosuppressive events. Therefore, cases of infection without disease may occur even in New World primates [10, 34, 35]. Although *T. gondii* isolates from Brazil are generally known to be more genetically diverse and more virulent in mice [24, 34, 35, 36, 37, 38, 39], Pena et al. [27] isolated *T. gondii* by mouse bioassay and performed molecular detection by PCR‐RFLP and genotyping using 10 genetic marks in a Howler monkey (*Alouata belzebul*, Parvorder Platyrrhini) that died in Parque Estadual Dois Irmãos zoo, in Recife, Pernambuco, Brazil. The isolate (TgRhHmBr1) was not lethal to the infected mice in the bioassay and had already been described in chickens and in a goat from the same region of Brazil. This highlights the need for further studies on *T. gondii* isolation and strain virulence in Brazilian primates.

In this study, the only individual that showed clinical signs, suggestive of toxoplasmosis, had been kicked out from the troop, which is a strong stressor. The event occurred two days before the animal started showing clinical signs of toxoplasmosis and was characterized by frequent aggressions from the dominant male of the troop and others from its coalition. The male was separated from the group and temporarily housed alone in a lesser enclosure at the out of exhibit area of the zoo and two days after the separation, the clinical symptoms started. The clinical signs observed in this animal were compatible with those described in cases of fatal toxoplasmosis in humans and non‐human primates [4, 11, 12, 13, 14, 17, 22], and the PCR‐positive diaphragm sample further supports the possibility that death was due to toxoplasmosis.

In the present study, a reduction in antibody titers was observed in two animals examined on multiple occasions. This reduction may have resulted from immunological stabilization associated with the animals' adaptation to the environment, given their recent introduction to the troop. Despite this decrease, the titers remained high, reaching 12 800 at the time of the last sampling. In the case of the male *S. flavius* (#58516), which died during the experimental period, antibody titers decreased from 25 600 to 12 800 between the first and final samplings, with the latter performed at the time of death. Because death occurred shortly after the animal was separated from the troop, placing it under high stress, it is difficult to determine that the titer decrease, once no data are available on titer progression between the two sampling points. The relatively sudden death may not have allowed sufficient time for the immune system to respond by producing antibodies.

The female *S. flavius* (#50494), which also underwent serological testing on two occasions, showed an increase in antibody titer, from 12 800 to 25 600; however, she did not exhibit any clinical signs compatible with toxoplasmosis. It is worth noting that this female was geriatric and occupied the lowest hierarchical rank among the females in the group. She also had been isolated from the troop, a situation that likely caused substantial stress and may have influenced her immune response to the infection. The only animal that maintained a stable antibody titer throughout the study period was an *S. libidinosus* (#05214), which belonged to a troop that had been housed at the zoo for many years and had well‐established hierarchical rankings.

This study supports previous reports of anti‐*T. gondii* antibody seropositivity in Neotropical primate species in the absence of clinical signs of toxoplasmosis. It underscores the importance of monitoring this infection in these animals and highlights the need for appropriate management within Institutions, since the introduction of new animals is relatively frequent and adverse reactions from established troops can compromise the animals' welfare and influence their immunity, leading to the onset of clinical toxoplasmosis.

## Funding

This work was supported by Fundação de Amparo à Ciência e Tecnologia do Estado de Pernambuco (FACEPE), Agência Estadual de Meio Ambiente de Pernambuco (CPRH), Parque Estadual de Dois Irmãos (PEDI) and the Superintendency of Instituto Brasileiro do Meio Ambiente e Recursos Naturais Renováveis, Pernambuco (IBAMA‐PE), and Conselho Nacional de Desenvolvimento Científico e Tecnológico (CNPq).

## Conflicts of Interest

The authors declare no conflicts of interest.

## Data Availability

The authors declare that the data comprising this article can be accessed upon request to the respective data holders. Raw data regarding test results are available in spreadsheets and can be requested from the corresponding author. Raw data concerning animal history, behavioral records, necropsy reports, and any other data related to the animal husbandry routine used in this study must be requested from the zoo administration via the e‐mail address gtmf@semas.pe.gov.br, as these data are part of the technical archives of Parque Estadual Dois Irmãos Zoo.
